# What is the significance of the traditional pinching mode of holding chopsticks?

**DOI:** 10.1186/s40101-020-00223-z

**Published:** 2020-05-04

**Authors:** Yoshihiro Shimomura, Takeaki Ohsawa, Megumi Shimura, Yali Xia, Koichi Iwanaga, Tetsuo Katsuura

**Affiliations:** 1 Graduate School of Engineering, Chiba University, 1-33 Yayoi-cho, Inage-ku, Chiba City, Chiba 2638522 Japan; 2 Graduate School of Science and Technology, Chiba University, Chiba, Japan

**Keywords:** Chopsticks, Manipulation, Electromyography, Motor learning, Non-dominant hand

## Abstract

**Background:**

The purpose of this study was to clarify the influence of manipulation mode of chopsticks on the learning process, using assessment of task performance and electromyography, and to understand the significance of the traditional manipulation mode from the viewpoint of physiological anthropology. Previous studies have described two modes of manipulating chopsticks, the traditional pincers-pinching mode and the scissors-pinching mode.

**Methods:**

We conducted experiments with two conditions of holding chopsticks: scissors mode and pincers mode. Eight subjects participated and were assigned to these modes, and they learned handling tasks in their assigned mode for 5 days with the non-dominant hand. We measured task execution times and conducted electromyography of the following muscles: first dorsalis interosseus, flexor pollicis brevis, flexor digiti minimi brevis, flexor digitorum superficialis, and extensor digitorum.

**Results:**

The training effects were found in each mode. The pincers mode showed significantly shorter task performance times than did scissors mode. On electromyography, significant increases in activity of flexor digiti minimi brevis and tended an increase in flexor digitorum superficialis and a decrease in extensor digitorum occurred in pincers mode but not in scissors mode.

**Conclusions:**

The traditional mode of holding chopsticks was associated with not only high task performance but also an advantage in terms of learning motor control.

## Background

**Fig. 1:**
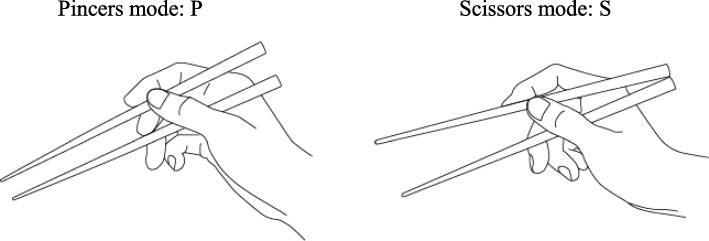
Modes of holding chopsticks. In the P modes, the pivot point is at the tip of the thumb. In the S mode, the top and bottom chopstick are touching like scissor arms, and there is no fixed pivot point at the tip of the thumb

Chopsticks have a long history, having been used in China since the fourth or fifth century B.C. [1, 2]. These utensils are used by more than 1.5 billion people around the world, mostly in East Asia, and their shape, material, and method of production differ with each country’s unique culture [3]. As with other Western-style utensils, the relationship between shape and manipulation performance have been studied [1, 4, 5]. A clear difference between chopsticks and other hand tools is that there are different ways to hold chopsticks [6–11]. Methods of holding chopsticks are largely divided into the traditional pincers-pinching (P) mode and the scissors-pinching (S) mode [6–8, 10] (Fig. 1). In general though, it can be said that all users of chopsticks use either P mode or S mode, with approximately half using one mode and half the other [4, 6–8]. When someone is gripping a chopstick with one hand, they grasp it with their index finger, middle finger, and thumb. P mode can secure another chopstick in place because the movement of the upper chopstick is independent. However, the movement of both chopsticks is not considered to be entirely independent in S mode because the upper chopstick is not held like a writing instrument. In this way, P mode leverages the advanced coordination of the human hand so that independent force can be applied to both chopsticks. The traditional P mode has been shown to have higher operational performance than S mode [4, 7]. However, aside from ergonomic operational performance, the only reasons why P mode is considered the “correct holding method” are the superior aesthetic appearance and the fact that this mode is customary [4, 5]. Under these circumstances, it may not therefore be appropriate to define P mode as correct [8, 10, 12].

Studies have been conducted on use of chopsticks as a motor task from the viewpoint of motor learning and hand dominance [13–16]. Several studies also have attempted to assess chopstick manipulation with electromyography [6, 17]. However, the relationship between mode of holding chopsticks and training or learning remains unclear [4]. Some studies have focused on use of chopsticks and human development and learning [6, 9–12]. While identical twins may be similar in their ability to use chopsticks or lack [11] thereof due to similar anatomical characteristics of the musculoskeletal system, motor learning regarding how to use chopsticks may be achieved by increased efficiency of muscle activity [17–19] which is an individual process that begins through the observation of others [12]. The mode of chopstick usage acquired after birth through motor learning may be one expression of whole body coordination [20].

Various functions that enable use of chopsticks are possible thanks to the ability of the unique human hand to manipulate complex tools [21, 22] and motor learning function. When questioning the significance of mode of holding chopsticks, it is necessary to consider the viewpoint of physiological anthropology [20]. The present study thus aims to clarify the effect of mode of holding chopsticks through motor learning in the non-dominant hand in order to investigate the significance of the traditional mode as the first study of chopsticks in physiological anthropology.

## Methods

### Subjects

Subjects for the study were eight Japanese university students (six male and two female) who provided consent after receiving a detailed explanation of the experiment. All were right-handed. Subjects were assigned to each holding mode in half. No significant differences were found between the groups in grip strength, hand length, hand width, or the distance between the outstretched thumb and middle finger.

### Tasks

All subjects performed the following training tasks once a day for 5 consecutive days with the assigned mode (P or S). The training period was set at 5 days because learning to manipulate chopsticks with the non-dominant hand is possible in a few days to a week [17]. Based on previous studies on ergonomics [4–6], five tasks were selected that enabled subjects to practice basic manipulation of chopsticks, precise grasping, and forceful grasping. Tasks for learning basic manipulation were a simple open and close task (once a second for 1 min) and a simple pinching task to stably pick up an object (wooden barrel-shaped cylinders with a base diameter of 10 mm, maximum diameter of 18 mm, and height of about 30 mm; performed 10 times). Tasks to learn precise grasping were a reciprocal moving task that involved moving the above objects back and forth between trays (three sets of moving five objects back and forth between the left and right tray for a total of 15 times) and a build-up task that consisted of making stacks of three of the objects (three times). The forceful grasping task was a mixing task in which a highly viscous liquid was mixed (starch syrup was mixed clockwise and anticlockwise at 60 cpm for 30 s each time).

### Measurements

To assess task performance, the execution time required for the reciprocal moving task and build-up task was measured each day. On the first and last day of the experiment, muscular activity was measured in the following muscles with electromyography: first dorsalis interosseous, flexor pollicis brevis, flexor digiti minimi brevis, flexor digitorum superficialis (in the indicis side and in the digitus minimus side), and extensor digitorum. The MP-150 data acquisition system and AcqKnowledge 3.9 data analysis software (BIOPAC Systems, Inc., Goleta, CA, USA) were used. The analog signal from TSD150 series active electrodes (on-board band pass filter 15–500 Hz, gain 330, input impedance 100 Mohm ) was set to 1000 Hz with 16-bit A/D conversion at an input range of ± 10 V. Data was recorded to a computer operating Microsoft Windows.

### Data analysis

For execution time, representative values used were the mean of three sets for the reciprocal moving task and the build-up task. Two-way analysis of variance was performed on the representative values with the number of practice days and chopstick holding mode as factors. For electromyography, root mean square (RMS) values were obtained while the subjects performed tasks, and paired *t* tests were performed on the values for the first and last day for each task and each chopstick holding mode. Probability values of less than 5% were considered significant and those of less than 10% were considered to show a tendency. The data was analyzed with Microsoft Excel and KaleidaGraph 4.0 (HULINKS, Inc., Tokyo, Japan).

## Results

**Fig. 2:**
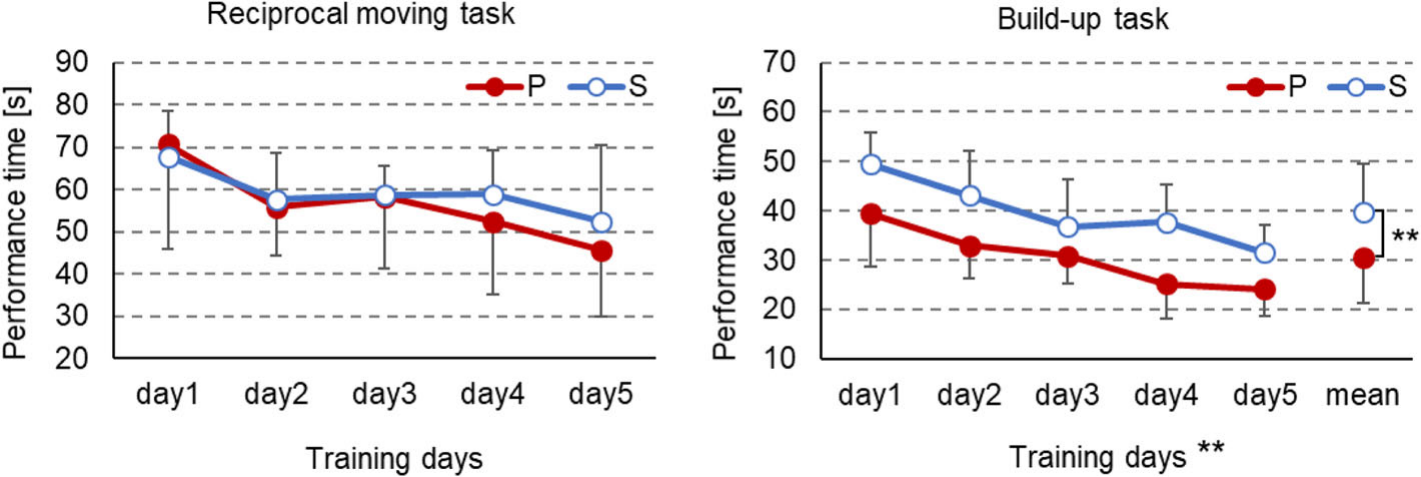
Task performance results. Shown as mean and standard deviation. **p < 0.01

**Fig. 3:**
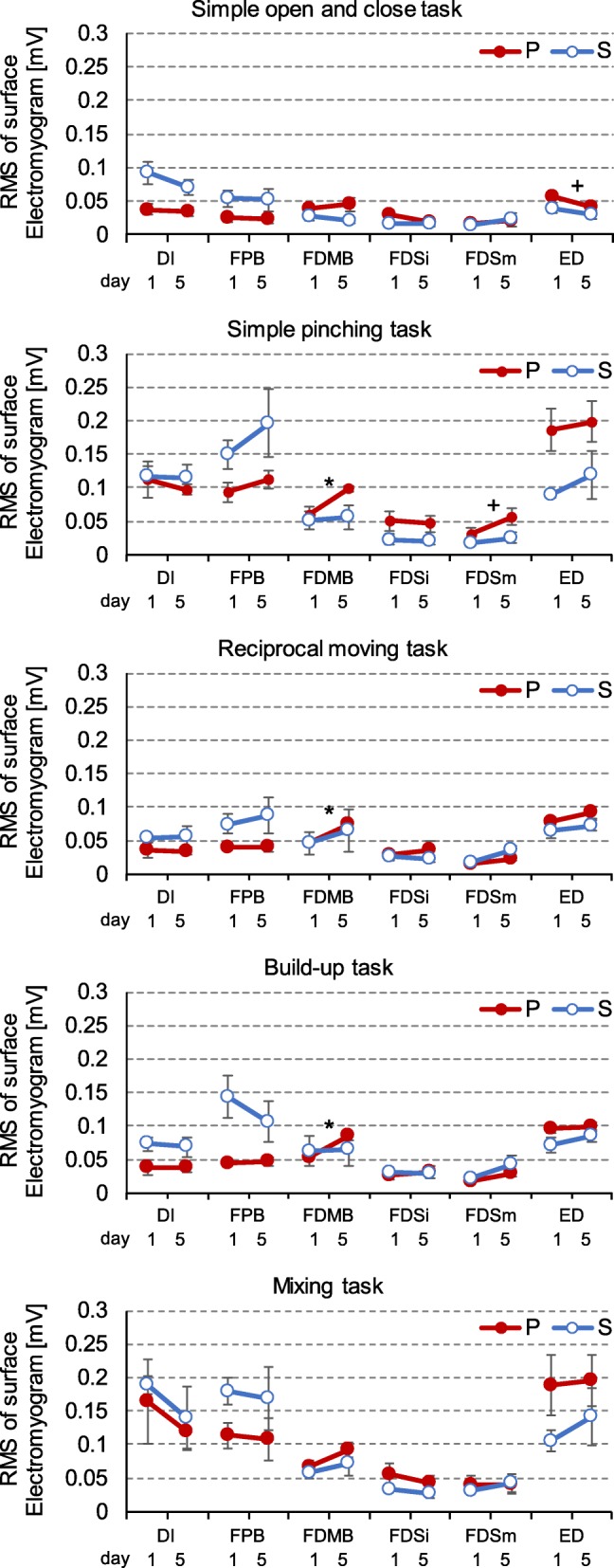
Changes in muscle activity during 5 days for each task and mode. Shown as mean ± standard error. Abbreviations of muscle names: DI: first dorsalis interosseous, FPB: flexor pollicis brevis, FDMB: Flexor digiti minimi brevis, FDSi: indicis side of flexor digitorum superficialis, FDSm: digitus minimus side of flexor digitorum superficialis, ED: extensor digitorum. *p < 0.05, +*p* < 0.1

Task performance results are shown in Fig. 2. No significant interactions were observed between number of days and mode for either task. The *F* values for the reciprocal moving task and build-up task were *F*(4, 30) = 1.40 and *F*(4, 30) = 4.30, respectively, and there were significant main effects of the number of training days (*p* <0.01) in the build-up task. In the build-up task, execution time was shorter for the P mode group than for the S mode group (*F*(1, 30) = 11.1, *p* < 0.01). Figure 3 shows the changes in muscle activity over the 5 days. In the P mode group, activity level increased significantly in flexor digiti minimi brevis and showed some tendencies of an increase in flexor digitorum superficialis and a decrease in extensor digitorum. In the S mode group, no tendency and significant changes were seen for any task or muscle.

## Discussion

In both modes of holding chopsticks, a training effect was seen in the non-dominant hand [7, 17] with one of precise grasping tasks over a short, 5-day period [17]. In a build-up task that required precise grasping, those using the P mode showed high performance, supporting previous studies [4, 7]. Motor learning here generally involves optimizing the co-contractive activation of muscles through repeating the experience, and increasing performance level [18, 19]. Increased activity in flexor digiti minimi brevis in the precise grasping task shown by the P mode group acts to stabilize the underside of the chopsticks as a synergist, thereby increasing performance. The tendency of changes in activity in flexor digitorum superficialis and extensor digitorum represent the increase in agonist muscle activity and decrease in antagonist muscle activity that occurs when grasping something with chopsticks. No tendency and significant changes were seen in muscle activity in S mode, suggesting that there was no electromyographic evidence of motor learning in this study. This may be because the finger control is functionally simple due to its holding style [4]. These experimental results show the facilitation of motor learning during chopstick use [6, 17] is easier in P mode, but not in S mode.

Nevertheless, the reason why mode of holding chopsticks has become polarized into P and S modes, and its cause of motivation, are still unclear. Learning to use chopsticks begins with observing the actions of another individual, often a parent [12]. Muscle activity is then made more efficient with growth and practice [17–19]. Whether or not the user acquires the P mode may be related to whole body coordination [20] and based on anatomical characteristics [22] or neuro-muscular adaptation [23] as well as the lifestyle and culture [4, 5, 8, 12] of the user. P mode, which is considered the traditional mode for holding chopsticks, does not only show higher task performance than S mode—it may also be the mode that actively brings out the latent functional potentiality related to motor learning, based on coordination between the hand and the brain in humans. It still remains unknown how chopstick users are able to sense these characteristics and why they consider the P mode to be correct and aesthetically pleasing.

## Limitation

The current study uses standard Japanese chopsticks; however, it does not take into account different characteristics such as the shape, weight, or surface friction of these chopsticks. It is therefore necessary going forward to examine the effects of the physical properties of chopsticks on how they are held.

## Conclusion

Through evaluating task performance and performing electromyography during different modes of holding chopsticks, the present study showed that the traditional mode is associated with high task performance and possible facilitation of motor learning.

## Data Availability

The datasets used for the current study are available from the corresponding author on reasonable request.

## Abbreviations

P: Pincers pinching mode
S: Scissors pinching mode
RMS: Root mean square
DI: First dorsal interosseous muscle
FPB: Flexor pollicis brevis muscle
FDMB: Flexor digiti minimi brevis muscle
FDSi: Indicis side of flexor digitorum superficialis muscle
FDSm: Digitus minimus side of flexor digitorum superficialis muscle
ED: Extensor digitorum muscle
